# Dispersal syndromes of *Vachellia caven*: Dismantling introduction hypotheses and the role of man as a conceptual support for an archaeophyte in South America

**DOI:** 10.1016/j.heliyon.2023.e17171

**Published:** 2023-06-09

**Authors:** Nicolás Velasco, Ramiro Bustamante, Christian Smit

**Affiliations:** a Conservation Ecology Group, Groningen Institute for Evolutionary Life Sciences, University of Groningen, Groningen, the Netherlands; b Departamento de Ciencias Ecológicas, Instituto de Ecología y Biodiversidad, Facultad de Ciencias, Universidad de Chile, Chile; c Charles Darwin Research Station, Charles Darwin Foundation, Santa Cruz, Galápagos, Ecuador; d Cape Horn International Centre, Cape Horn County, Chilean Antarctic Province, Chile

**Keywords:** Acacia caven, Long-distance dispersal, Hydrochory, Zoochory, Trans-Andean

## Abstract

*Vachellia caven* has a disjunct distribution at the southern cone of South America, occupying two major ranges: west of Andes (Central Chile) and east of them (mainly the South American Gran Chaco). For decades, the species has been subject to various ecological and natural history studies across its distribution, but questions concerning its origin in the western range remain unresolved. Thus far, it is unclear whether *Vachellia caven* was always a natural component of the Chilean forests, and "how" and "when" the species arrived in the country. In this study, we revised the dispersal syndromes of the species and contrast the two main hypotheses of dispersion to the west of Andes that have been proposed in the 90's, namely animal versus human-mediated dispersal. For this, we reviewed all scientific literature on the species and explored the available information on morphology, genetics, fossil records and distribution patterns of closely related species. Here we illustrate how the collected evidence provides support for the human-mediated dispersal hypothesis, by including a conceptual synthesis that summarizes the outcomes of different dispersal scenarios. Lastly, and regarding the positive ecological effects this species has in the introduced area, we suggest reconsidering the (underappreciated) historical impacts of archaeophytes and rethinking the role that indigenous human tribes may have had in the dispersion of different plants in South America.

## 1 Introduction

**Fig. 1:**
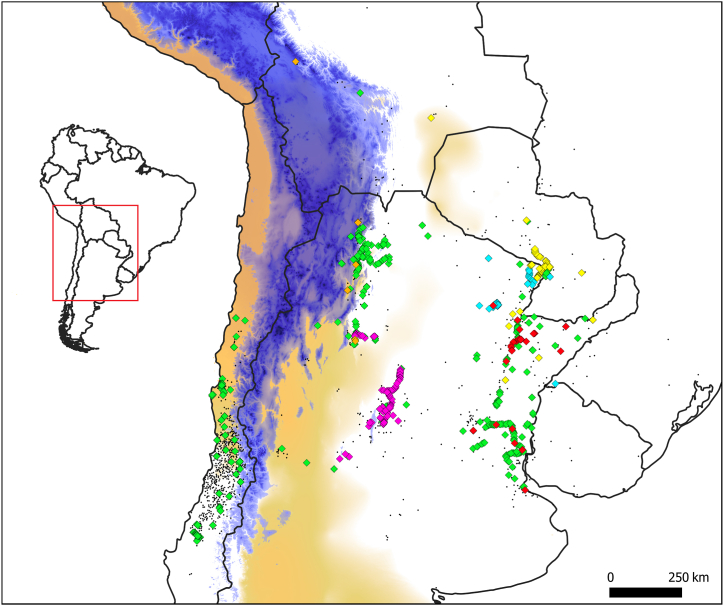
The distribution of *Vachellia caven* in Southern America. Each color represent an infraspecific variety: green = var. *caven*; pink = var. *dehiscens*, orange = var. macrocarpa; cyan = var. *microcarpa*; red = var. *sphaerocarpa*; yellow = var. *stenocarpa*. Georeferenced records without infraspecific taxonomic identification are denoted as black dots. The elevation of the Andes mountains higher than 2000 m.a.s.l. is depicted in blue (source: [134]), with the darker the color the higher the altitude. In light brown the South American Arid Diagonal is shown (source: [135]), with the darker the color the drier the conditions. *Vachellia caven* presence data comes from GBIF [136] and CONAF [137].

*Vachellia caven* is a Fabaceae tree that occurs in different climates across the southern cone of South America [1]. The species distribution displays a disjunct pattern, with two large biogeographic regions separated by the Andes mountain range: i) the eastern range, mainly across the Great South America Chaco, and ii) the western range, mainly in Central Chile. The Andes is a geographic barrier with altitudes higher than 3000 m.a.s.l, and widths ranging from 100 km in the southern latitude (∼32° S), up to 400 km in the northern latitudes of the Altiplano region [2]. In these settings, *V. caven* generally does not overpass altitudes beyond 1000 m.a.s.l [3] and rarely reaches up to 2000 m [4]. Additionally, following the Andes, the South American Arid Diagonal (SAAD) constitutes an additional barrier that constraints species distributions by exerting a xeric effect along a homogenous band of approximately 400 km in width [5] and which only few species can surpass. *Vachellia caven* is scarcely present inside the boundaries of the SAAD, unable to surpass its effects beyond northern Chile or Argentinian Patagonia (Fig. 1).

*Vachellia caven* is currently considered a native component in the Chilean flora and has shown positive effects on the Mediterranean-type plant communities [[6], [7], [8], [9]], yet some studies have suggested that the species may be non-native, thus explaining the disjunct distribution [4,7,[10], [11], [12], [13], [14]]. For example, in the western distribution, the phenology of *V. caven* is similar to that of eastern subtropical species, being a deciduous tree, and producing flowers before shoots in the growing season [11]. Both features are atypical for the Mediterranean Chilean flora as most species are evergreens that produce shoots before flowers.

In present times, *Vachellia caven* seeds are predominantly dispersed by cattle, specially in the western range [15]. However, it is a challenge to elucidate which was the dominant species responsible for *V. caven* dispersal prior to the introduction of these relatively new herbivores in the 16th century [16]. It is also challenging to argue a mechanism that does not include animals, as these are well-known as the current disperser, but it seems at least, that in some parts of the distribution (specially at the eastern range) water may be a possible candidate. Based on the assumption that today's ecological and evolutionary traits were shaped by previous dispersal agents, two dispersal syndromes have been proposed: hydrochory and endozoochory, with the former the least discussed in studies to date. The dispersion unit in *V. caven* is the whole fruit (Fig. S2) making water a potentially important dispersal mechanism [4,17,18]. However, the problem with accepting hydrochory at least for the western range, is that there *V. caven does* not occur in many places with flooding-drought dynamics (such as rivers). In addition, in many western localities, the species establish on hill slopes which makes water an implausible dispersal mechanism. Nevertheless, in the driest localities in Chile, such as the Atacama Desert, the species follows dry river banks. Thus, even if the species grows in dry-like places in the western distribution, it may still have some past traits related to hydrochory.

**Fig. 2:**
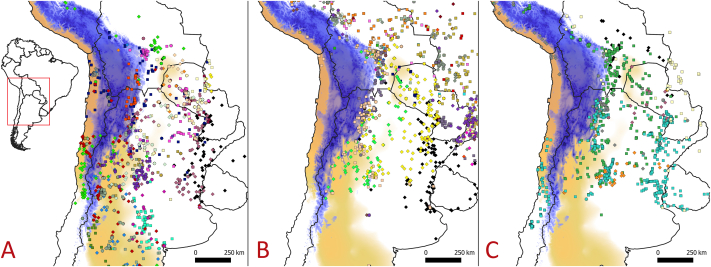
Distribution patterns of the most common native Fabaceae in the *Vachellia caven* distribution range. Panel A = distribution for *Prosopis* species; panel B = distribution of *Senegalia* species; panel C = distribution *Vachellia* species. Last two genera correspond to the old taxonomic *Acacia s.l* group. In each panel, colored diamonds or squares represent a different species (Species list on Fig. S4). The elevation of the Andes mountains higher than 2000 m.a.s.l. is depicted in blue (source: [134]), with the darker the color the higher the altitude. In light brown, the South American Arid Diagonal is shown (source: [135]), with the darker the color the drier the conditions. Presence data from GBIF [107,136,138].

Another pattern that may suggest an introduction event is that Fabaceae species are not common on both sides of the Andes (Fig. 2). *Prosopis* species, for example, have been filtered by the Andes mountains and even if they exist in both regions, only a few species occur in Chile, of which some are even endemic [19]. The case of *Vachellia caven* is even more striking, as it is one amids more than 20 species of closely related taxa (Acacia *s.l*) distributed in Argentina [[20], [21], [22]] but the only species occurring widely in Chile (Fig. 2) [23,24]. Only two other related species occur, *V. macrantha* and *Senegalia visco;* however, its occurrences are rare in Chile, limited only to boundaries on the extreme north desert.

Hence, the origin of *V. caven* in Chile is unknown, but a couple of potential hypotheses have been suggested that involve dispersal by animals or humans [7,10]. The aims of this study are: i) summarise and propose the expected processes involved in the distribution pattern of the species (vicariance vs long-distance dispersal, i.e. split by geographic barriers or transported along large distances), ii) examine the *V. caven* dispersal syndromes (i.e. hydrochory vs endozoochory), iii) explore the different trans-Andean dispersal hypotheses proposed for the presence of the species in Chile (namely: guanaco/animal hypothesis vs human-mediated hypothesis), and contrast and update them with the most recent and relevant scientific literature, to finally argue which hypotheses are the most parsimonious through a conceptual synthesis.

Although the most recent taxonomic revision has divided Southern American *Acacia s.l* into *Vachellia* and *Senegalia* [25] (Fig. S1), the current taxonomic treatment for *V. caven* has not yet accounted for the variability within the species that was published previously under *Acacia caven* (Fig. 1). For that reason, and depending on the reviewed literature, we will use *Vachellia* and *Acacia* interchangeably through this study. For a complete understanding of the ubiquity of *V. caven* on the phylogeny of *Acacia s.l* also see Clarke et al. [26], and Gómez-Acevedo et al., [27].

## 2 Methods

The references mentioning ideas about the status of *V. caven* in Chile, or including possible dispersion mechanisms or the main trans-Andean dispersal hypothesis, were included as pivotal point for this review. These correspond to the 14 more important research articles addressing these problems (Table S1). After identifying main topics related to *V. caven* history discussed in these 14 sources, the scheme for this review was divided in sections addressing each individual theme. The topics selected were two related to patterns: “phylogeny and fossil records” and “varietal patterns”, two related to dispersion mechanism: “hydrochory”, “endozoochory”, and four addressing trans-Andean dispersal hypotheses: “the guanaco hypothesis”, “other vertebrates’ hypothesis”, “limitations on animal dispersal hypotheses”, and “the human-mediated hypothesis”. For each topic we made an independent scoped review searching for information supporting it or against it. Each topic was screened through Google Scholar, by doing a search with complementary words or synonyms (e.g., for “varietal patterns”, words as *morphology* OR *varities*), and including words “*Acacia caven*” or “*Vachellia caven*”. The topics that did not had sufficient information in the literature specifically at the species level, were broaden at the genus or family level (e.g., fossil records). Topics related to specific animals or dispersal limitations, were broaden also reviewing them across South America or in the whole Andes, without incorporating interactions to *V. caven,* but priorizing in general other legume species (e.g., other *Vachellia* species, or the *Prosopis* genus). Literature was screened looking at the titles and abstracts to assess its initial suitability, and considered until 2021 (the year in which the research was made). Considering the pivotal literature (Table S1), this review concluded in 133 sources selected, which included 89 research articles, 18 reviews, 6 perspectives articles, 11 book chapters, and 4 theses. We also included grey literature for this review, specifically five reports. Finally, four datasets were used to represent figures.

## 3 Evidence for possible processes: vicariance vs long-distance dispersal

Disjunct patterns mainly result from two different processes: i) by *vicariance,* when the advent of a geographic barrier splits a species distribution, and ii) by long-distance-dispersal, i.e. when a species is capable of surpassing geographic barriers by movement beyond the normal crossing range of the organism [28,29]. The final result of both processes is that new isolated populations arise that could differentiate from the original populations over time. To unravel which of these processes explain the occurrence of *V. caven* in Chile, the first step is to consider its distribution in South America.

### 3.1 Phylogeny and fossil records

Given the separate distribution by a mountain range, one may assume that vicariance is responsible for the split. In this context, the proposed phylogeny for *Vachellia* group suggests that *V. caven* originates around 10 My BP [30], hence, crossing a time-span when the Southern Andes had not yet entirely arisen [31]. The current distribution of the more closely related species, *Vachellia farnesiana* and *Vachellia tortuosa* [27]*,* are both North and Central American, while a few occurrences of the former are present in the eastern part of South America (Brazil). This could indicate a probable split between *V. farnesiana* and *V. caven* in the dry habitats of eastern South America; a region that is also seen as a possible centre of diversification for other legumes such as *Prosopis* species [19,32]. Nevertheless, there is no support for a scenario where *V. caven* was paleo-present in the western range or that it rapidly dispersed into Chile during these periods since the fossil records of the species do not support its presence in the country before the total rise of the Andes. Some related fossil woods of *Acaciaxylon* from the Eocene (∼2.5 My BP) are present in Mocha Island (a Chilean island about 40 km from the continent) [33]. However, today the record is less suitable because a name change has been proposed and *Acaciaxylon* is no longer recognised due to relatedness to another non-close Fabaceae subfamily (Fig. S1) [i.e., *Dalbergia* woods, Faboidae, [34]]. Interestingly, a related fossil record of pollen from the Late Miocene/Early Pliocene in Navidad (∼33–34°S) [35] may suggest the presence of *Acacia* species in the western region. Yet, it forms only weak support for *V. caven*, because the record was designated as *Acaciapollenites* without further description or affinity to an extant species. Other western palynological records for these periods are available for a few *Acacia s.l* species from other subgenera (*Heterophyllum*) that are naturally no longer present today in Chile [36]. So, diagnosed records do not support a potential distribution of *V. caven* in Chile through these paleo periods. In general, *Acacia* records are uncommon in palynological-sediments studies worldwide because its large pollen (poliads) is difficult to be dispersed by wind [37]. However, despite the scarce records west of the Andes, in the eastern distribution, there are frequent palynological records of related Fabaceae throughout the Eocene to the Pliocene, mainly in Brazil and Argentina [38]. Interestingly, this data is also congruent with fossil woods for the same period [39]. Those palynological and fossil wood patterns are in concordance with today's *Vachellia* and other legume tree distributions in the rest of South America (Fig. 2). With the use of molecular markers from *Vachellia* spp. and its related mutualist ants (*Pseudomyrmex* spp.), other phylogenetic analyses propose a more recent diversification of the *Vachellia farnesiana* clade (in which *V. caven* is part (Fig. S1)), around 6 My BP [27], reducing the likelihood of an Andean vicariant event for *V. caven*.

In more recent historical times (Quaternary) and east of the Andes, *Acacia*-records (excluding anthropogenic collecting sites) are abundant since mid-Holocene [40,41], with palynological evidence of the presence of *V. caven* in the late Pleistocene as far east as Uruguay [42]. All these records are from semi-continuous periods and not from rare events in lacustrine sediments cores. In contrast, at the western distribution, there are no natural records (i.e. not from anthropogenic or culture settlements collecting sites), even in locations where the species is abundant today [[43], [44], [45]]. All in all, from the above, we can conclude that there is more empirical support for a historical long-distance dispersal scenario than for a vicariant process for *V. caven*.

### 3.2 Varietal patterns

Additional support for a long-distance dispersal scenario comes from morphological studies conducted on the species. Various studies report the presence of morphological varieties in the eastern distribution [4,20,21,46,47], and indicate that six morphological varieties can be discriminated based on fruit traits such as form and size (Fig. S2), and vegetative traits such as the number of leaflets. While all these varieties occur in the eastern distribution, only one variety (namely *A. caven* var. *caven*) has been documented for the western distribution (i.e. Chile, Fig. 1) [4]. Most of these studies have focused on the eastern populations, while the western populations are underrepresented in these comparisons. Nevertheless, they seem to support that the western morphology is not a novelty compared to the eastern range. Aronson [4] made the only study that sampled Chilean populations, but even that does not formally compare the morphology among "var. *caven*" populations of the western and eastern distribution.

According to ecotypic measurements of *V. caven* there is evidence of regional differentiation. Seedlings from eastern subtropical provenances have different survival, growth, frost tolerance [48] and cyanogenic production [10] than other seedlings from the eastern distribution. Nonetheless, the western provenances used in these studies do not seem to differ on these ecotypic measurements from other used eastern provenances [11,48]). In the last decade molecular analyses using RAPD and AFLP markers have been performed in Argentina, elucidating that varieties are also genetically distinct, even among different habitats within a same variety [1,47,[49], [50], [51]]). For the Chilean populations, however, such genetic analyses have thus far never been performed.

Jordano [29] summarizes and discusses the different terminology used for long-distance dispersal. The author frames that *sensu stricto* long-distance dispersal is considered when species fall outside the boundaries of two spatial reference frames, namely the geographic and the neighbourhood (genetic) domain. In our view, the evidences from the available *V. caven* studies are in line with this definition and support the long-distance dispersal hypothesis, and not the vicariant scenario. Yet, new morphological and molecular studies are needed for an in-depth comparison and clarification of the relationships between western and eastern populations.

## 4 Local dispersal syndromes & germination

### 4.1 Hydrochory

Dispersion of *V. caven* fruits has been pointed out as feasible through water, indicating hydrochory as a potential dispersion syndrome, specially for the eastern distribution [4,17,18]. At its eastern distribution, there is a clear pattern where the species is present in areas with high summer rainfall (e.g., around the flooded areas in the wet Chaco), whereas the species is absent or less abundant in dry areas (dry Chaco and Pampa). Even if *V. caven* can establish in dry areas it has been considered a phreatophyte species with roots in regular contact with subsoil water [52]; similar to other Fabaceae tree species [53]. The variety "*dehiscens*" is the only variety with open pods when mature and is present in the driest areas of the eastern species distribution. This means that, for this variety, released seeds are left to be dispersed by birds, ants or rodents [4,54]. The other varieties are all indehiscent, meaning that the pod/fruit does not split or open to release seeds. Considering that the fruit mesocarp is spongy or forms air cavities that envelope the seeds [4] (Fig. S3), the indehiscence is a remarkable trait that confines seeds in light pods. Furthermore, the hard and impermeable coat of the seeds makes them imperishable after several days underwater which in fact, even stimulates their germination, a feature that would harm other legumes [55]. All of the above adds to the fact that the fruit ripens in the rainy season. Thus, dispersion through lakes or rivers has been recorded at some places in wet ecoregions of the eastern distribution [pers. obs.; [3,18,56]]. Remarkably, the distribution and change of three *V. caven* varieties (var. *stenocarpa*, var. *microcarpa* and var. *sphaerocarpa*) from small-elongated to big-round fruit morphs, falls into the wet Chaco ecoregion and follows the development of the Paraná River from its source up to the estuary at the Atlantic Ocean (Fig. 1) [1,4].

An important argument that can point to water as the main dispersal mechanism in the past is that *V. caven* is highly recognised to grow in perturbed areas on both sides of the Andes. In Argentina, other *Vachellia* species (e.g., *V. aroma*) are more common in native forests, growing together with other xeric or subtropical species. In contrast, *V. caven* is strikingly present where some level of disturbance reduces competition with other forest species (pers. obs.). This raises the question as to which natural environmental disturbances have this species adapted to? Wildfire is a possible candidate, but for Chile it is generally accepted that vegetation did not evolve under fires regimes [57]. Co-evolution with fire makes sense on parts of the dry Chaco [58], but its influence may be weak since *V. caven* is distributed mainly in flooded areas in the eastern range. The only frequent and broadly distributed disturbance of the eastern range were summer floods which were common on the Quaternary [59]. Marine introgressions through the Miocene [60,61] may add part of the explanation for the eastern distribution pattern of the species. In summary, it is possible that flooding disturbances are related to the ecological traits of *V. caven* by creating suitable conditions for the dispersal of the pioneer species to colonise new soils and ravines.

### 4.2 (endo)zoochory and animal-related traits

The hard coat of many legume seeds is seen as a trait to resist chewing and smoothen the passage through the gut of large animals. The current *V. caven* endozoochorous dispersion by cattle, frequent in the western range, is explained as an analogy of past plant-animals interactions [62] with similar effects on African *Acacia* species by today's large herbivores [63]. For example, without a pre-germination treatment to soften the seed coat, less than 5% of *V. caven* seeds reach germination [64,65] Propagation techniques of *V. caven* include enhanced germination protocols where seeds are submerged in sulphuric acid at high concentrations (up to 2 h in 98% [H_2_SO_4_]) [66]. This is commonly understood as an adaptation to acids found in the gut passage of herbivores. However, commercial concentrations have far higher pH values than that found in animals [67]. The high percentages of germination obtained by commercial protocols (up to 90%) are significantly higher than achieved by gut passage and therefore some studies do not agree that ingestion by cattle enhances germination of *V. caven* seeds [67,68]. The studies that do claim positive effects of cattle ingestion show only slightly improved germination of *V. caven* seeds (i.e. from ∼1% to 6%) [15,69]. Hence, this suggests that cattle would merely act as a disperser, not as a ‘germination enhancer’.

**Fig. 3:**
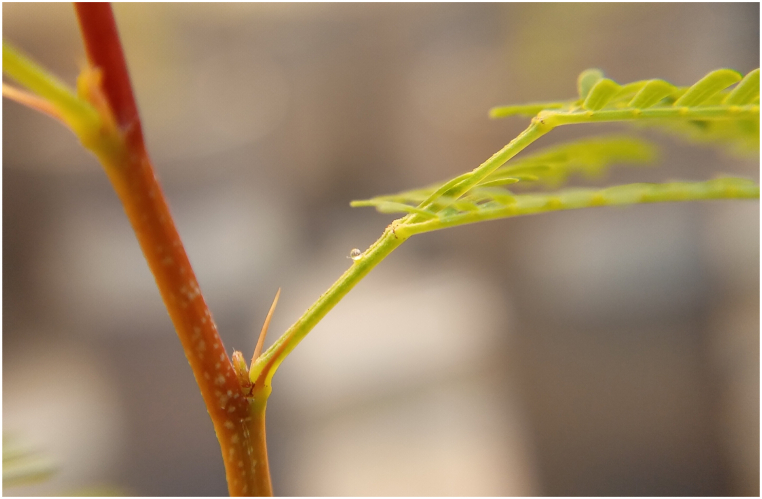
–Seedling of *V. caven* exuding ant rewards through the petiolary gland.

While the traits thus far discussed can relate to endozoochory by herbivores, they may also be seen in another context: namely the animal-interaction with its principal seed predator. Seeds are vital organs that secure future species survival, and the hard seed coat of *V. caven* can also be seen as a trait that co-evolved in response to predation by *Pseudopachymeryna spinipes*. This bruchid beetle species is found across the whole *V. caven* distribution and is the major contributor to seed mortality [[70], [71], [72]]. Most of the bruchids’ attacks happen when pods and seeds are immature [73] to avoid the hardening of seed coats. Another trait of co-evolution in response to predation is the presence of thorns that have been associated with protection against large browsers [74]. The presence of thorns in *Acacia* species is not only related to the direct protection against large herbivores, but the thorns in *Acacia s.l* are also known to benefit spiders and nesting birds [75], which in return protect trees from phytophagous insects. In addition, many *Acacia s.l* species have a mutualistic relationship with ants [76] in which the ants provide protection for the tree from other animals. This is not only evident for swollen thorns species (i.e., ones with big and hollow thorns) which ants uses as nesting sites, but even the unswollen thorny *acacias* have exudative ant-rewarding glands (petiolar glands in *V. caven*) (Fig. 3).

Other common traits mentioned to cope with herbivory are cyanogenic compounds [10,77] and compensatory growth [74], but can be equally related to herbivory by small herbivores. Thus, traits usually stated as arguments for adaptation to large herbivores and indirectly leading to zoochory dispersion may not be necessarily ecologically related.

Notwithstanding that both hydrochory and endozoochory dispersal syndromes may be plausible mechanisms for local dispersion of the species, only endozoochory suits for a long-distance dispersal from east to west because both distributional ranges are not connected through water pathways. So, from here on, the focus will be on biotic long-distance hypotheses.

## 5 Trans-andean dispersion hypothesis

While some studies suggest that extinct megafauna may have had effects on the dispersion of *V. caven* to Chile [4,62] there is no empirical support for this idea. If extinct megafauna had played a role, the presence of *V. caven* would have been expected in Chile long before the Holocene epoch. The Megafauna, among which included stegomastodonts and giant sloths, became extinct across South America in the Late Quaternary Extinction episode (Pleistocene-early Holocene) by a combination of climatic and vegetation changes and the indigenous man [78]. If megafauna would have been important dispersers, the populations of *V. caven* would have shown contracted distribution ranges due to limited dispersal distances, or have shown some alike stepping-stone distributions. Although there is little evidence for historical dispersal of *V. caven* to Chile by extinct megafauna, multiple other hypotheses have been proposed that include dispersal by extant fauna.

### 5.1 The "guanaco" (*Lama guanicoe*) hypothesis

One common idea in the scientific literature concerning the long-distance dispersion of *V. caven* is dispersion by camelids ‘guanaco’ (*Lama guanicoe*) [79], which effectively cross the Andes through corridors [80]. The idea is sustained in its effect may resemble the current effects of cattle [15]. The ‘guanaco’ hypothesis has been proposed in several studies and is probably the most common dispersal hypothesis for the appearance of *V. caven* at the west of the Andes [7,13,62,69,74,81,82]. Some authors refer to "Fuentes et al. [83],” as evidence for the guanaco hypothesis; however, this study assessed the effects of cattle and goats while guanacos are only briefly discussed as a possible analogue. Others instead refer to another study [69], as an "experiment of guanacos in semi captivity". However, this seems again incorrect referencing, because Fuentes et al. [69], studied Chilean vegetation structure while nothing is specifically stated on guanacos. Experiments with "guanacos in semi captivity" are described in Fuentes & Simonetti [84] and Simonetti & Fuentes [85,86] where the diet preferences of guanacos and other animals were studied by offering different species of shrubs of the Chilean matorral. Interestingly, while *V. caven* was not included in any of these studies, the researchers discussed the low interaction between guanacos and shrubs, stating that guanacos act more as grazers than browsers, a behaviour also mentioned in other studies [87]. In fact, guanacos tend to prefer herbs of low height rather than taller species [88]. In another study [79] authors added that the guanacos in semi captivity ate and dispersed *V. caven* seeds, although reported as “Fuentes & Simonetti, unpublished results”. Foraging of guanacos on *V. caven* was only included years after the gestation of the guanaco hypothesis [89], yet that study did not include predation of fruits or seeds by guanaco, but in leaves instead. More recent support for the role of guanacos comes from faeces piles of hybrid or captive guanacos with seeds or seedlings of *V. caven* [90,91]. Yet, as *V. caven* seeds could have been present in the soil prior to the defecation events, additional experimental studies are needed for confirmation.

Ingestion of *V. caven* pods by guanaco's probably occurs together when feeding on their target grasses and herbs, especially considering that under *Vachellia* spp., grass and herb biomass tends to be higher [7] thus making it an attractive foraging location. However, based on the nutritional quality of the fruits, *V. caven* fruits only seem to offer a very small reward. The hard-ligneous pericarp and the scarce mesocarp do not compare with the rich fleshy pulp of other Argentine *Acacia s.l* and legumes. For example, guanacos foraging in Argentina where *Vachellia* spp. co-occurs had leaf tissues in their faeces [92] but consumption of *V. caven* pods was not reported. In fact, guanacos show a marked preference for other leguminous species such as *Prosopis* sp., *Geoffroea decorticans* and *V. aroma*, all species with fleshy and sweet fruits [20,93]. Hence, in the eastern distribution, *V. caven* does not appear as a standard part of the guanaco's diet. The aforementioned trends find also support in past conditions, as coprolites (i.e., fossilized faeces) in Argentina show that herbs are by far the most consumed item in Camelids' diets [94] while Fabaceae are almost inexistent. Another issue that undermines the relative importance of guanacos for *V. caven* dispersal is the low abundance of these mammals in major parts of their historical distribution where they were extirpated almost entirely from central Chile by indigenous man [80]. In summary, there is little evidence that guanacos forage on *V. caven* pods and this is too weak to sustain a trans-Andean dispersal event.

### 5.2 Hypothesis of other vertebrates

Besides the already discussed guanaco's, other vertebrates could also have contributed to long-distance dispersal. Aronson [4] suggests that the Greater Rhea (*Rhea americana*) is among the possible dispersers of *V. caven*. The greater Rhea has a distribution consistent with that of *V. caven* on the eastern side and subspecies of this bird have distributions pattern which overlaps to some degree with each of the *V. caven* varieties [95,96]. Similar to guanacos, there is little evidence that Fabaceae seeds pass through the guts of Rheas [97]. Moreover, data on the interaction between rheas and *V. caven* is weak, with reported foraging on the species being scarce, even in localities where *V. caven* is abundant [98]. Although the Greater Rhea is not present in Chile and its role in the dispersion of *V. caven* is uncertain, its sister species the Darwin's Rhea (*R. pennata)* may have played a role. The species is currently confined to southern and northern Chile, Argentina, and southern Peru and Bolivia [95] with its present distribution at high altitudes or in pampa areas which hardly overlap with *V. caven*. Yet, quaternary records for this species are located in areas as distant as Buenos Aires for late Pleistocene and Holocene periods [99], or even as recent as 1000-700 BP in Córdoba [100], where they probably shared the same distribution as *V. caven*. For the western region, in past periods they may have co-occurred in the valleys of the Atacama region or areas of Chilean-Argentine Andean corridors to the latitudes between north/central Chile [99]. Nonetheless, the Darwin's Rhea subspecies (*Rhea pennata* subsp. *tarapacensis*), of Northern Chile did not distribute in any other country, which makes it unlikely that this bird contributed to trans-Andean dispersion from east to the west of Andes [95,96]. Considering the improbable dispersion of *V. caven* to Chile by vertebrates such as rhea and guanacos, the dispersal by other large vertebrates is equally unlikely.

Considering medium-small-sized vertebrates, the only potential candidates might be birds, as they move farther than mammals or reptiles. Yet, only a few species are shared between Chile and Argentina, as many species in the former (and overlapping with *V. caven* distribution) are endemic [101]. Potential medium size passerines *Turdus falcklandii* and *Curaeus* only inhabit the southern part of Argentina (e.g., Patagonia), where *V. caven* is not present, and other species, such as *Mimus thenca* have been present in Argentina apparently since a few decades only [102]. Notwithstanding, the groups of parrots could be a more potential candidate considering their size and beak structure, which in turn could feed on larger and harder seeds. Species of the genus *Enicognathus* are in part sympatric with *V. caven* in the western range, yet, they only inhabit austral Argentina, in the province from Chubut/Neuquén to the south, where *V. caven* is not present [103]. The parrot *Cyanoliseus patagonus* is split into subspecies separated by the Andes, and its differentiation has already been assessed, addressing that connectivity between both ranges is really low [104]. In addition, through studying its phylogeographic structure has been proposed that eastern populations all descend from one introductory event from the west in the late-Pleistocene [105]. The only interaction documented with *V. caven* is seeing them transporting branches but in localities far from the Andes, such as Córdoba [106]. Also, these movements only reached 6 km/day and were for constructing nesting in specific areas. All in all, this suggests a low likelihood of a trans-Andean introduction by birds.

### 5.3 Limitations of the animal-dispersal hypotheses

Even when considering guanacos, rheas, and other (mega)vertebrates as dispersers of *V. caven* to Chile, we encounter the following difficulties.

1) The pattern of variability (or richness) of *Vachellia species* is too abrupt between east and west Andes (i.e., only one infraspecific variety of one species in the western distribution, while several species in the eastern distribution). If animals are responsible for the arrival of *V. caven* to Chile, it would be likely that the other more rewarding *Vachellia* species be dispersed as well (e.g., *V. aroma*, *V. atramentaria*). Such is the case for other Fabaceae species which have fleshy and rewarding fruits and for which many species are indeed shared between both regions (e.g. the *Prosopis* genus: [93,107]). For these, similar large herbivores' dispersal hypotheses have been proposed [19].
2) During the late Pleistocene – Holocene transition, intermittent cooling events between warming periods after the Last Glacial Maximum (LGM) [78], would strengthen the barrier effect of the Andes. As *V. caven* has requirements of a subtropical species, it could be expected that the distributions on both sides have been more faraway, widening the distance between regions compared to the present.
3) A significant problem is the distance for the migratory voyage. Past endozoochory by megafauna (including *L. guanicoe*) is thought to reach dispersal events up to 6 km, while the time of seeds retained in guts varied between 0,5 and 3 days [108]. Today, some guanacos individuals can migrate up to 80 km and have home-ranges up to 163 km^2^ in central Chile [109]. But the majority of guanaco populations in Mendoza show that the mean distance between summer and winter foraging plains is around 35 km annually [110]. Movement distances of wild guanacos after stress events (e.g., capture for live-shearing) are up to 17 km/day and maintained only for two days [111], while regular daily movement distances are around 3–9 km. At present, the smallest distance between the two *V. caven* distributions is around 165 km (Los Andes locality – Mendoza city), meaning that the dispersal voyage must be done in a short time across the Andean barrier, weakening its contribution to the dispersion hypothesis to the west.
4) Finally, large sections of the Argentine-Chilean Andean corridors do not fit the ecological requirements for *V. caven* (i.e., average annual temperatures are too cold), and even if there were suitable Andean corridors, one would expect to see a nucleated distribution pattern following endozoic diffusion (i.e., stepping stone dispersal in patches) [19], which is not the case. Burkart [19] also suggested that the presence of endemic *Prosopis* spp. at each side of Andes demonstrates few animal connections between both regions, reducing the importance of animals as trans-Andean dispersers.

All in all, the animal-dispersal hypotheses limitations, provide a simple summary of why a common idea in literature may not necessarily be the most parsimonious idea to consider as an explanation for *V. caven* presence west of the Andes.

### 5.4 The "human-mediated" hypothesis

**Fig. 4:**
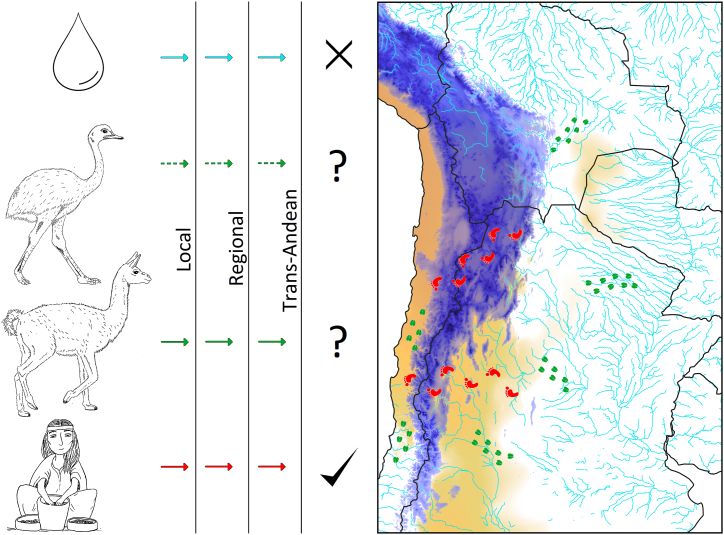
Conceptual synthesis of the dispersion syndromes and dispersion ranges. Hydrochory (cyan), endozoochory (green), human-dispersed (red). Each color has different outcomes at surpassing local, regional or trans-Andean barriers. Solid lines represent probable scenarios, while dashed lines represent uncertain scenarios. Humans are the most likely trans-Andean scenario compared to biotic dispersers, here represented by rhea and guanaco. Dispersion ranges are illustrated by humans (red) and animals (green) footprints, respectively. The “X” represents an impossible scenario. Elevation of the Andes mountains higher than 2000 m.a.s.l are indicated in blue (source: [134]), with the darker the color the higher the elevation. The South American Arid Diagonal is indicated in light brown (source: [135]), with the darker the color the drier the conditions.

Ovalle et al. [7], proposed that indigenous people are probably responsible for the presence of *V. caven* west of the Andes, arguing that cultural and commercial trade existed between Argentina and Chile during the Holocene. Indeed, trans-Andean passages used for indigenous trade are well documented [112,113]. Accordingly, this hypothesis would overcome most encountered problems with the hypotheses of animal dispersal; not so much the transfer of seeds via digestive systems, but rather the transport of seeds by humans (i.e., in pockets or pots) would have been the main dispersal mechanism (Fig. 4). Trade between regions by indigenous groups might have been quite common as the reconstruction of vegetation before the European conquest suggests. In fact, there is evidence that the Chilean Mediterranean zone was already transformed into matorral under influence of the ca. 10^6^ indigenous people that already lived there [114]. Historical literature points out that the first Spaniards noticed that indigenous people used *V. caven* as medicine, as coffee equivalent and as a fabric dye [7,115], usages that are still present on both sides of the Andes [116,117].

It is known that between South American countries, other legume species were traded in ceremonial rites or as food during the Holocene (e.g., *Anadenanthera colubrina*) [118]. For *Prosopis chilensis,* which also shows a disjunct distribution among Chile, Bolivia and Argentina, the populations found in the former are considered to originate from transhumance [119]. Recent genetic analyses support this idea, indicating little genetic diversity in Chile coming from both other countries [120]. Additionally, after the Peruvian pepper tree (*Schinus areira* L.), *V. caven* is the second most widespread tree species in extra-tropical South America [10]. This connection is interesting, as also for this Peruvian pepper tree, Inca transportation has been proposed as a cause for its current distribution [121].

The trans-Andean human dispersal hypothesis also finds support in the lack of adequate fossil records before the Holocene in Chile and the widely invariant morphology of pods in Chile. Heusser [122] found a few pollen grains of *Acacia* species in places as far as the Llanquihue Lake (∼41°S). Even if these are not a specific proxy of our species, Heusser used *V. caven* as a taxonomic affinity descriptor to name the pollen. The data are in support of a Holocene introduction, with postglacial dates between ∼9400 and 7800 years BP. Interestingly, this record was found very close to the Monte Verde archaeological site (∼30 km), which is considered as one of the earliest human occupations in South America [123].

*V. caven* fossil records in Chile are closely associated with indigenous settlements during the Holocene [[124], [125], [126]], appearing between ∼2500 and ∼700 BP. Some of the records correspond to the Aconcagua culture, which used the similarly named Aconcagua Valley in Central Chile [124]. The passages through the Andes which connected the "Norte Chico" regions of Chile with the provinces of Mendoza, San Juan, and Catamarca in Argentina were used by the Aconcagua, Inca and Diaguita cultures and following human occupations as well as during the Spaniard conquest [113,124]. Even today, these passages are the most crucial routes of trading between Chile and Argentina.

The human-mediated hypothesis surpasses several limitations that the other hypotheses have (i.e., animal-related hypotheses and even hydrochory mechanism) (Fig. 4). The human-mediated hypothesis has the most supporting evidence, and it seems the most probable one given the species’ morphological and historical patterns. Although we acknowledge that the other dispersion syndromes by other biological vectors may have played a (small) role, they do not explain long-distance dispersal events. Moreover, it is important to realise that these are not necessarily mutually exclusive. For instance, man could have had a role in the movements of big animals, as guanacos and rhea were used as food resources for the indigenous people [127]. New ecological and molecular studies on the differentiation of *V. caven* between both ranges are needed to clarify its distribution pattern and population expansion times in Chile.

## 6 Synthesis

The introduction of *Vachellia caven* to Chile seems to correspond to the late Pleistocene – early Holocene, most likely via human-vector dispersion from the east of the Southern Andes of South America. This would mean that *V. caven* is an "*archaeoaphyte*" species, in accordance with the concepts discussed by Preston et al. [128],: "exotic species, introduced during previous modern times (1492 CE)". Similarly, *V. farnesiana,* a sister species of *V. caven*, is now seen as a historical invasion in Australia, brought about by long-distance dispersal over the seas via humans during the Holocene [129]. Here, we propose a historical context for the presence of *V. caven* in Chile, a species that also has a significant positive ecological effect on its environment. Plenty of studies indicates that this species has a positive role on the plant communities inhabiting the western range [6,8,9,[130], [131], [132]]. Considering its ecological importance, some authors have rejected the idea of *V. caven* being an introduced species, also accounting for the negative impact the labelling of ‘introduced species’ could have for society [81,90]. We agree that integrating both the ethical and scientific considerations, *V. caven* should remain treated as a native species. Yet, for a better understanding of the history and biology of invasive taxa, we suggest that some degree of freedom should be accepted on definitions. Richardson et al. [133], suggest that for the definition of biological invasions, one should only look at the ecology and geographic/demographic status of species and not focus on whether these species are harmful to the community. In this context, *V. caven* has all the traits that could correspond to an old invasion event: it was human-assisted and has surpassed a set of geographic and ecological barriers, thus colonising new areas and establishing new populations. In addition, this historical invasion has had a positive effect on the recruitment of sclerophyllous communities. Perhaps it is time to rethink the ecological outcome of introduced taxa and their historical contexts, and particularly the role of indigenous tribes as shapers of biodiversity.

## Author contribution statement

All authors listed have significantly contributed to the development and the writing of this article.

## Data availability statement

No data was used for the research described in the article.

## Declaration of competing interest

The authors declare that they have no known competing financial interests or personal relationships that could have appeared to influence the work reported in this paper.
