# Supplementary material for: Dispersal syndromes of Vachellia caven: Dismantling introduction hypotheses and the role of man as a conceptual support for an archaeophyte in South America
Source: Heliyon. 2023 Jun 9;9(6):e17171. doi: 10.1016/j.heliyon.2023.e17171 (PMC10276236; doi:10.1016/j.heliyon.2023.e17171)
Supplement: Multimedia component 1 [file mmc1.docx]

**Supplementary material**

**Supplementary Table 1 – Seminal and more important literature addressing questions about status, origin and dispersion of *Vachellia caven*.** For each source the main evidence is given. A=animal dispersion, H=human dispersion, W= water dispersion (hydrochory). S = in support of; O = in opposite of. Lowercase letter indicates weak evidence. Question mark indicates a potential support.

| **Year** | **Authors** | **Study** | **Evidence** | **A** | **H** | **W** |
| --- | --- | --- | --- | --- | --- | --- |
|  |  |  |  |  |  |  |
| 1981 | Gutiérrez, J. R., & Armesto, J.J. | El rol del ganado en la dispersión de las semillas de *Acacia caven* (Leguminosae). *International Journal of Agriculture and Natural Resources*, 8(1), 3-8. | Cattle ingestion (proxy of vertebrates) | S |  |  |
| 1989 | Aronson J. & C. Ovalle | Report on a study of the natural variability, biogeography and potential for genetic improvement of *Acacia caven. Bulletin of the International Group for the Study ofthe Mimosoideae* 17: 111-121. | The first time the question regarding about origin of *V. caven* in Chile is introduced. Slightly mention as the probable recent introduction. |  | ? |  |
| 1990 | Fuentes, E. R., Avilés, R., & Segura, A. 1989 | Landscape change under indirect effects of human use: the Savanna of Central Chile. *Landscape Ecology*, 2(2), 73-80. | Often used in the literature to support *L. guanicoe* interaction with *V. caven.* Yet, the study includes this idea only as an analogue of goats and cows; animals really tested in the study. | S |  |  |
| 1990 | Ovalle, C., Aronson, J., Pozo, A., & Avendano, J. | The espinal: agroforestry systems of the mediterranean— type climate region of Chile. *Agroforestry Systems,* 10(3), 213-239. | Introduce the idea of *Lama guanicoe,* or humans moving seeds across the Andes. Also, suggests the option of domesticated ungulates. | S | S |  |
| 1992 | Aronson, J. | Evolutionary biology of *Acacia caven* (Leguminosae, Mimosoideae): infraspecific variation in fruit and seed characters. *Annals of the Missouri Botanical Garden:* 958-968. | Incorporates several potential dispersers, extant and extinct, as well as a potential dispersion through water bodies. | S |  | S |
| 1994 | Aronson, J., Ovalle, C., Aguilera, L., & Leon, P. | Phenology of an'immigrant'savanna tree (Acacia caven, Leguminosae) in the Mediterranean climate zone of Chile. Journal of Arid Environments, 27(1), 55-70. | Includes several patterns (morphological, biogeographic, chemical, and phenological) to argue that Chilean populations might be a recent introduction. |  | S |  |
| 2002 | Holmgren, M. | Exotic herbivores as drivers of plant invasion and switch to ecosystem alternative states. *Biological Invasions,* 4, 25-33. | Briefly discuss the potential origin, contrasting influence late-Pleistocene animals or the guanaco hypothesis versus evidence of the late appearance of *V. caven* in archaeological sites. Suggests *V. caven* as exotic in Chile. | O | S |  |
| 2003 | Pratolongo, P., Quintana, R., Malvárez, I., & Cagnoni, M. | Comparative analysis of variables associated with germination and seedling establishment for *Prosopis nigra* (Griseb.) Hieron and *Acacia caven* (Mol.) Mol. *Forest ecology and management,* 179(1-3), 15-25. | Evidence of high viability after a long period of seeds submerged in water. |  |  | S |
| 2011 | Van de Wouw, P., Echeverría, C., Rey-Benayas, J. M., & Holmgren, M. | Persistent *Acacia* savannas replace Mediterranean sclerophyllous forests in South America. *Forest Ecology and Management,* 262(6), 1100-1108. | Mentions a potential exotic origin and invasive status of *V. caven* in Chile, yet includes potential spread by domesticated *L. guanicoe.* | S | S |  |
| 2012 | Venier, P., García, C. C., Cabido, M., & Funes, G. | Survival and germination of three hard-seeded Acacia species after simulated cattle ingestion: The importance of the seed coat structure. South African Journal of Botany, 79, 19-24. | Evidence of low softened *V. caven* testa after simulated cattle ingestion | O |  |  |
| 2013 | Root‐Bernstein, M., & Jaksic, F. | The Chilean espinal: restoration for a sustainable silvopastoral system. *Restoration Ecology,* *21*(4), 409-414. | Sociological implications of considering *V. caven* as naturally dispersed (*L. guanicoe*) vs human-mediated dispersed. | S | O |  |
| 2016 | Root‐Bernstein, M. | Personal reflections on natural history as common ground for interdisciplinary multispecies socio‐ecological research. Geo: *Geography and Environment,* 3(1), e00015. | Directly presents both main hypotheses, one against the other. | S | O |  |
| 2017 | Root-Bernstein, M., Guerrero-Gatica, M., Pina, L., Bonacic, C., Svenning, J. C., & Jaksic, F. M. | Rewilding-inspired transhumance for the restoration of semiarid silvopastoral systems in Chile. *Regional Environmental Change,* 17*,* 1381-1396. | Feeding of *L. guanicoe* with *V. caven* branches | S |  |  |
| 2019 | Guerrero-Gatica, M., & Root-Bernstein, M. | Challenges and limitations for scaling up to a rewilding project: scientific knowledge, best practice, and risk. *Biodiversity,* 20(2-3), 132-138. | Discussion about *L. guanicoe* as a potential disperser of *V. caven*. Includes notes about *V. caven* germinating in faeces piles of the mammal. | S/o |  |  |


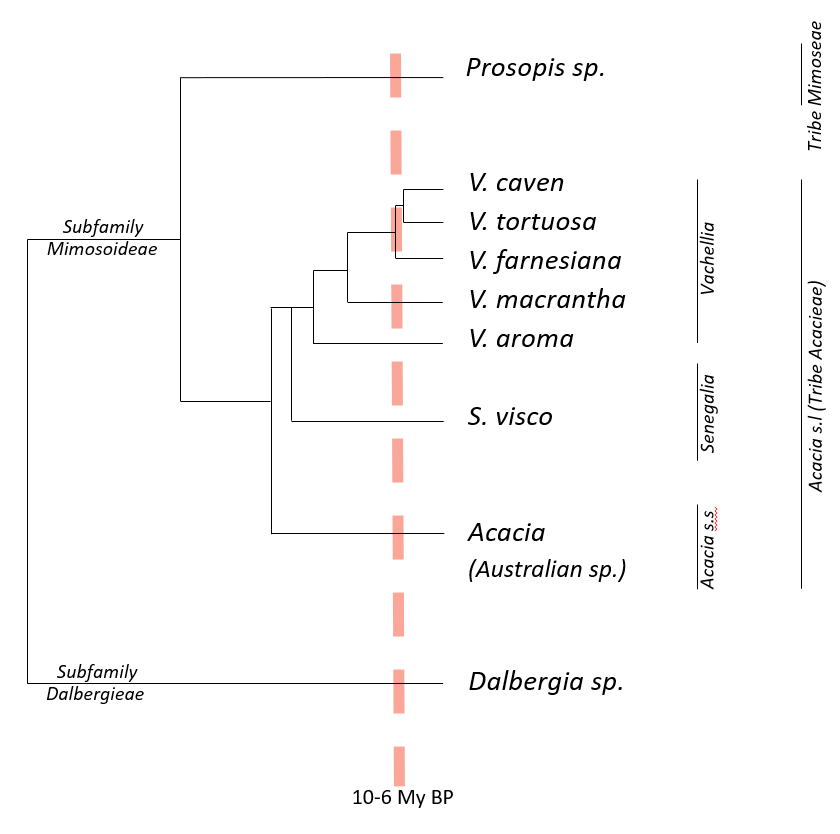


**Supplementary Figure S1 – Schematic relationship of some of the species on the Fabacea family, mentioned through the study.** New taxonomic treatments are included. The distances are not scaled.


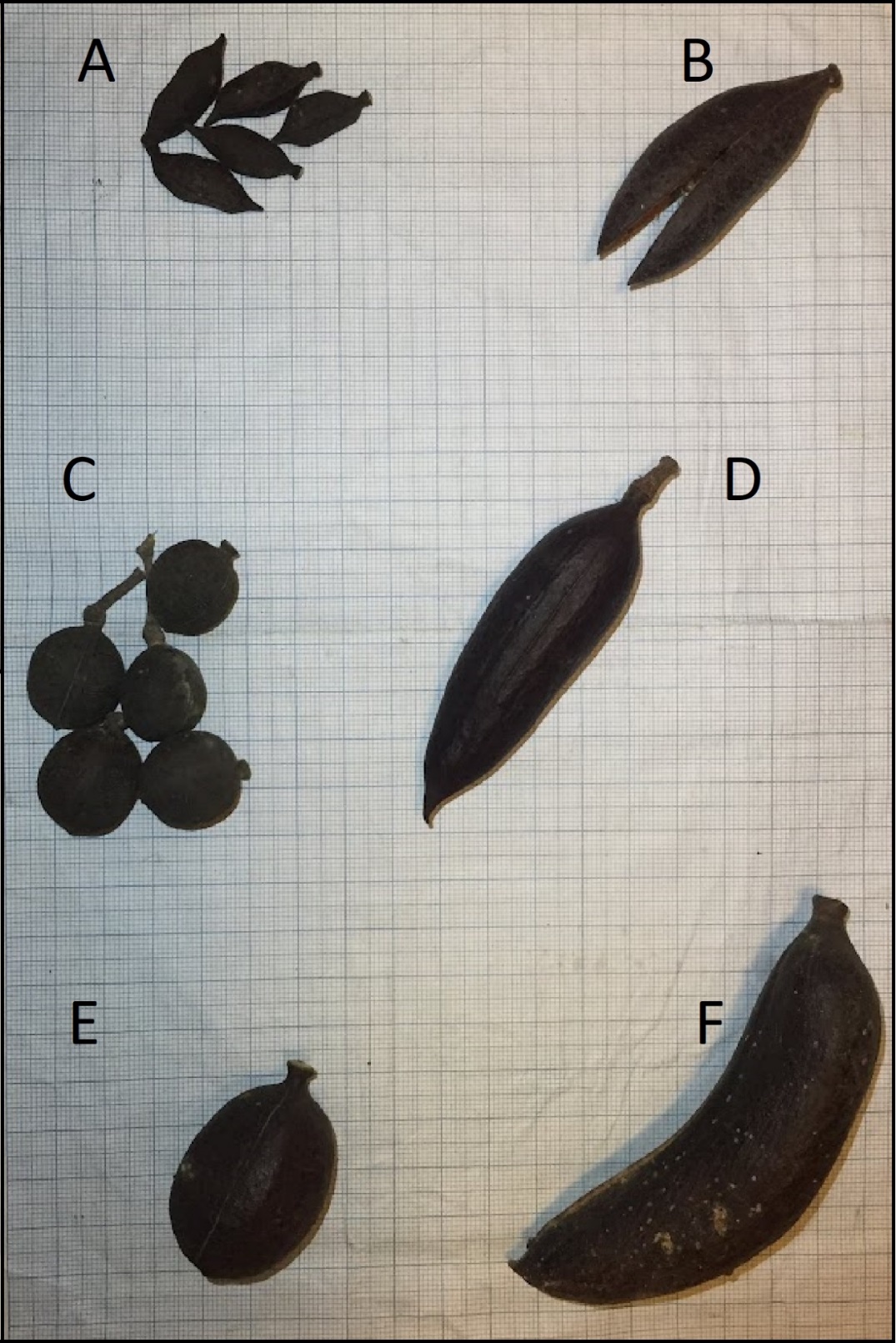


**Supplementary** **Figure 2 – Fruits of the specific varieties of the *Acacia caven* complex (i.e., *Vachellia caven*).** All fruit varieties together for size comparison, from top left to bottom right: a) var. *stenocarpa*, b) var. *dehiscens*, c) var. *microcarpa*, d) var. *caven*, e) var. *sphaerocarpa*, and f) var. *macrocarpa*.


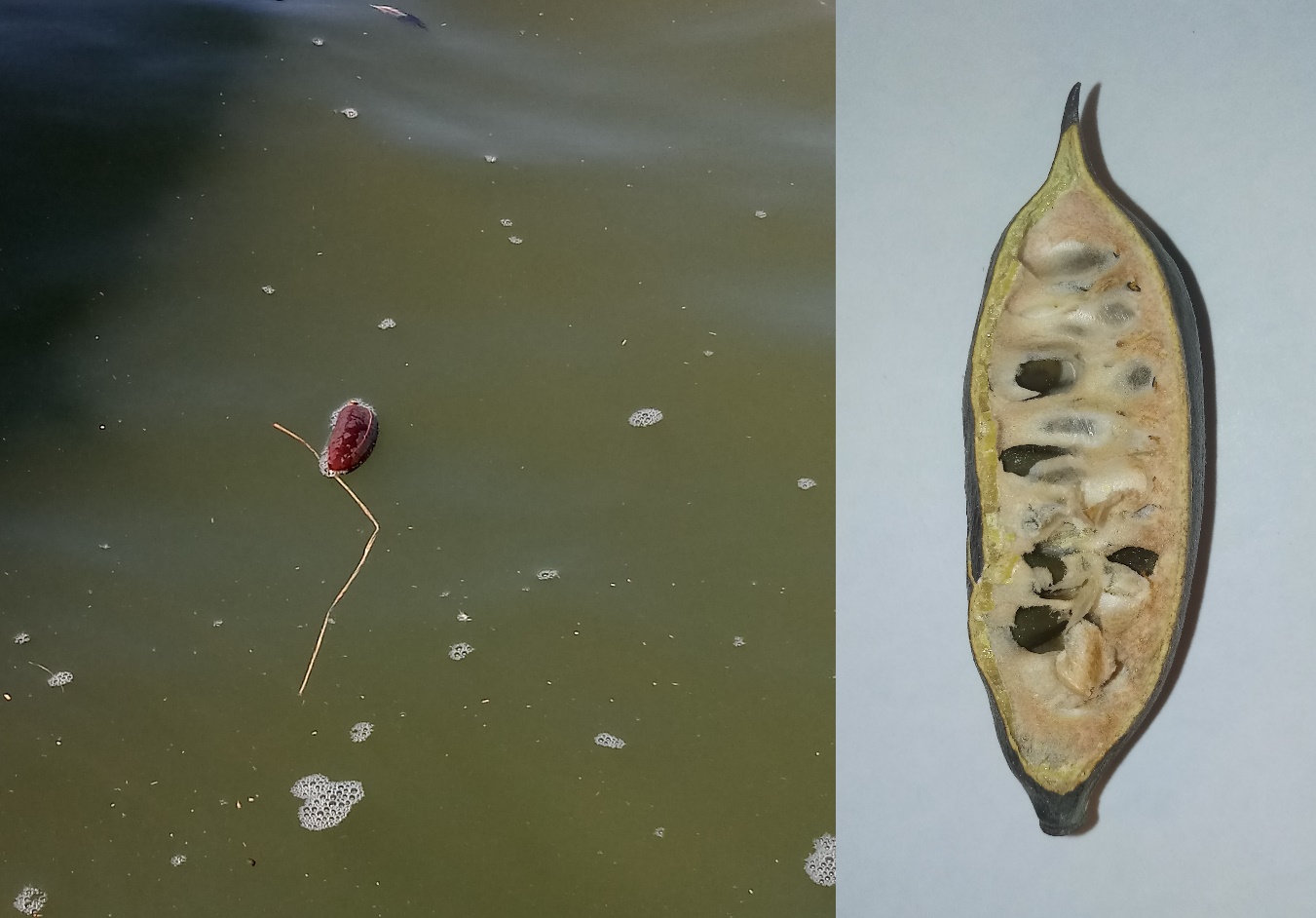


**Supplementary Figure 3 – Traits of *Vachellia caven* pod**. Left: fruit floating in a Carén lagoon (Chile), Right: spongy interior of a pod.


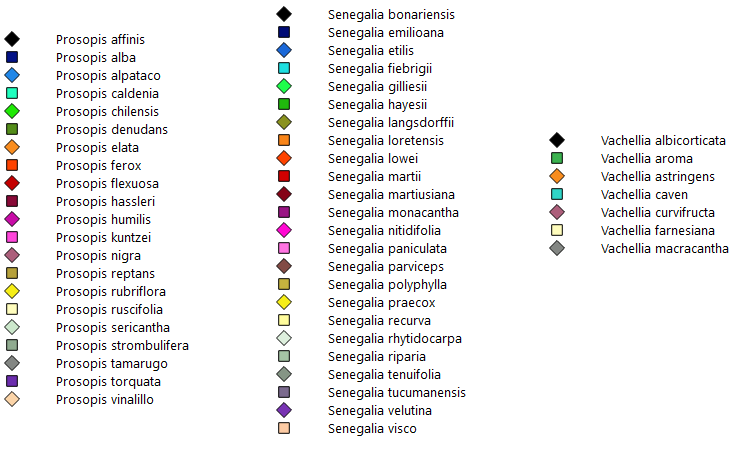


**Supplementary Figure 4 – Species list for *Prosopis*, *Senegalia* and *Vachellia* genus showed on Figure 2.**
